# An insight into the evolutionary history of Indonesian cattle assessed by whole genome data analysis

**DOI:** 10.1371/journal.pone.0241038

**Published:** 2020-11-10

**Authors:** Pita Sudrajad, Subiharta Subiharta, Yudi Adinata, Af’idatul Lathifah, Jun Heon Lee, Johannes A. Lenstra, Seung Hwan Lee

**Affiliations:** 1 Assessment Institute for Agricultural Technology–Central Java, Indonesian Agency for Agricultural Research and Development, Ministry of Agriculture, Kabupaten Semarang, Indonesia; 2 Beef Cattle Research Station, Indonesian Agency for Agricultural Research and Development, Ministry of Agriculture, Kabupaten Semarang, Indonesia; 3 Department of Anthropology, Diponegoro University, Semarang, Indonesia; 4 Division of Animal and Dairy Science, Chungnam National University, Daejeon, Korea; 5 Faculty of Veterinary Medicine, Utrecht University, Utrecht, Netherland; National Cheng Kung University, TAIWAN

## Abstract

The domestication of Indonesian cattle was investigated through a study of their genetic diversity, up to the genome level. Little documentation exists regarding the history of domestication of Indonesian cattle and questions remain despite a growing body of molecular evidence. In this study, we genotyped seven Indonesian cattle breeds using an Illumina BovineSNP50 Bead Chip to provide insight into their domestication and demographic history in a worldwide population context. Our analyses indicated the presence of hybrid cattle, with *Bos javanicus* and *Bos indicus* ancestries being most prevalent, as well as purebred cattle. We revealed that all the breeds were interconnected through several migration events. However, their demographic status varied widely. Although almost all the Indonesian cattle had an effective population size higher than the minimum level required to ensure breed fitness, efforts are still needed to maintain their genetic variability and purity.

## Introduction

Cattle movements after domestication were believed to be influenced by human migration patterns since cattle provided food and helped with crop cultivation [1–3]; however, the interactions between these factors remain unclear [4]. Of particular interest is how the breed diversity of cattle in Southeast Asia, and especially in Indonesia, was influenced by human migration patterns [5, 6]. Kikkawa et al. [7] suggested three possible ancestries for the admixture of the domestic cattle in this area, i.e., taurine, indicine and the local domesticated *Bos javanicus* which is also known as Bali cattle (BALI). The discovery of an ancient rectangular cattle bell and the narrative reliefs on Borobudur temple in–Indonesia depicting humped cattle with bell necklaces led to speculation that early Indian zebu were introduced to Indonesia at some time between the 9^th^ and 10^th^ centuries [5, 8–10], and that they were replaced by BALI in several regions [5], and crossed with BALI in other regions [6, 11, 12]. Documentation exists of the import of both taurine and zebu cattle into Indonesia during the Dutch colonial period from 1905 to 1920 [9, 11, 13].

Evidence of the hybridization between *Bos javanicus* and *Bos indicus* in Indonesian cattle has been confirmed by many scientists using varied approaches [4, 7, 9, 12–14]. The Ongole Grade (ONG), Madura (MAD), Jabres (BRE), Aceh (ACE) and Pesisir (PES) cattle were proposed as hybrids resulting from those crosses in Indonesia. Interestingly, a similar hybridization pattern was also identified for cattle in Thailand [15] and China [4]. How crosses between these two breeds (*Bos javanicus* and *Bos indicus*) occurred, and how they could give rise to many other breeds, remains unclear [14]. The most likely explanation for the diversity of Indonesian cattle breeds involves the variable level of zebu-BALI introgression that is admixed in each breed [4, 9].

Genome-wide single nucleotide polymorphism (SNP) data analysis is a powerful tool that can be used to investigate genetic variation related to the evolutionary history of cattle populations, since this approach uses a large number of markers that are widely dispersed and have diverged throughout cattle genomes [3, 4, 16–18]. The possible ascertainment bias that might emerge in population diversity analyses could be reduced if this approach is used [17]. In this study, we performed genetic diversity analyses of Indonesian cattle breeds using a BovineSNP50 Bead Chip (Illumina, San Diego, CA, USA) and predicted their population histories. We also included Kebumen Ongole (KBO) cattle in our analyses, which is a population from the southern coast of Central Java that has been reported to have superior phenotypic characteristics compared with the ONG population [19]. We hypothesized that there would be large genetic variation between KBO and ONG cattle.

To better understand the diversity of Indonesian cattle in their historical context, we added sample datasets from Thailand [15], Bangladesh [20], and other indicines and taurines from across the world obtained from Decker et al. [4]. This dataset was used as a reference in the population structure analyses to estimate the relationships between Indonesian cattle and the worldwide population. This study was conducted to address the following questions: 1) What is the genetic variation among Indonesian cattle breeds? 2) How did *Bos indicus* move from its origin to Indonesia over 1,000 years ago? 3) How did *Bos javanicus* become admixed with the other Indonesian, Thai and Chinese cattle breeds? 4) What is the origin of KBO and are they genetically related to other Indonesian cattle? And 5) What is an effective population size for Indonesian cattle breeds? We aimed to provide insights into the evolutionary history of Indonesian cattle populations by answering these questions.

## Materials and methods

### Ethical statement

The animal procedures related to our sample collections were approved under INSINAS activity No.6/INS-2/PPK/E4/2018 of the Indonesian Ministry of Research Technology and Higher Education. Genotypes for the remaining breeds were obtained from previously published data [4, 13, 15, 20].

### Animals and genotype quality controls

The animals used in this study were 247 individual heads of cattle composed of seven Indonesian cattle populations, i.e., BALI, MAD, BRE, ONG, KBO, ACE, and PES. In total, 107 samples were from our collections (samples from local breeding farms); 74 samples were supplied by Decker et al. [4], and 66 samples were supplied by Hartati et al. [13]. Images of the Indonesian cattle are presented in S1 Fig. An additional 490 foreign cattle samples from Thailand, Bangladesh and other worldwide populations were obtained from Wangkumhang et al. [15], Uzzaman et al. [20] and Decker et al. [4] (S1 Table).

Genomic DNA was isolated from blood samples of the Indonesian animals using the methods of Sambrook et al. [21]. DNA quantification was performed using a NanoDrop 1000 (Thermo Fisher Scientific Inc., Waltham, MA, USA). DNA samples were submitted for genotyping at a concentration of at least 20 ng/μl and 260/280 ratios > 1.8. Samples were genotyped for 54,609 SNPs using the Illumina BovineSNP50 BeadChip. The UMD 3.1 whole-genome assembly [22] was used to obtain genome coordinates of all available SNPs. The quality of the genome-wide data was controlled using SNP filtering in PLINK 1.9 [23] based on the following parameters: SNPs that fell outside of the Hardy-Weinberg proportions (P < 1e-04) and had a low call rate (< 90%) or a high rate of missing genotypes (> 10%) were not included. To reduce bias in the data, the minimum number of minor allele frequencies was limited to 1%. The BALI, ONG, MAD and BRE genotypes that were obtained from other sources [4, 13] were included in our dataset. Only genotypes from the autosomal chromosomes were used in further analyses.

### Diversity, population structure and phylogenetic analysis

Diversity and population structure analyses were completed using the following algorithms: 1) Inbreeding coefficient within-population (*F*_IS_) and pairwise fixation indices between-population (*F*_ST_) calculations [24], 2) heterozygosity and Nei’s genetic distance estimation [25], 3) genetic relationship matrix (GRM) estimation, 4) multi-dimensional scaling (MDS) analysis, 5) neighbor-joining trees, and 6) ancestral admixture prediction. The fixation indices, heterozygosity and Nei’s standard genetic distance analysis were completed using two R packages: Hierfstat [26] and StAMPP [27]. GRM was estimated using GCTA v1.25.2 [28]. A four-dimensional pairwise genetic distances matrix resulted from the calculation of the MDS algorithm in PLINK 1.9 [23] and was depicted as coordinates in R [29]. ADMIXTURE v1.23 [30] was used to detect possible ancestral mixtures of the populations using adjusted cluster models (K) of K2–25, shown as bar plots in R. To select a minimum error value for K, cross-validation analysis was performed. The neighbor-joining trees were constructed using SNPhylo [31] and depicted using FigTree v1.4.2 (http://tree.bio.ed.ac.uk/software/figtree/) [32].

### Migration events, linkage disequilibrium and demographic estimation

Extensive analysis of the relationships among cattle populations was carried out using TreeMix v1.12 [33]. This approach allows estimation of possible historical splits and mixes between populations, termed as migration events. A maximum likelihood tree of populations was created first. Two tree models were generated to identify both predicted ancestors (BALI and NEL) as the root, and to enable estimation of the migration patterns. To account for linkage disequilibrium (LD) in the construction of the trees, markers were grouped together in groups of 1,000 SNPs. Migration edges that best fit the data were evaluated based on the fraction of the variance defined in the matrix of the residuals, in which positive values were preferred. To identify possible traces of introgression in Indonesian cattle, we conducted *f3* statistical analyses introduced by Reich et al [34] using the *threepop* command line. Three-population statistical models (A, B and C) with a significant negative value for both the *f3* statistic and Z-score were selected as possible introgression events of populations B and C into population A.

The demographic history of the cattle population was reflected in the values of the estimated current and former effective population sizes (*N*_*e*_). *N*_*e*_ was estimated from the LD value using Sved’s equation [35], as explained by Sargolzaei et al. [36]. Before calculating *N*_*e*_, LD was annotated as *r*^*2*^ to measure the correlation of alleles at two loci [37]. We used the default PLINK 1.9 [23] and SNeP v1.0 [38] approaches to complete the estimations of LD and *N*_*e*_, respectively. The—*r2* command was used to obtain the LD value of SNP pairs and the—*ld-window-r2* was set to zero to obtain reports for all pairs. The historical *N*_*e*_ values were plotted using R following the estimated times in the horizontal ordinate.

## Results

### Population structure and diversity

In total, 52,886 autosomal SNPs were obtained from the 50K SNP dataset of our samples from Hartati et al. [13] and Uzzaman et al. [20], but we obtained only 42,885 and 38,650 autosomal SNPs from Decker et al. [4] and Wangkumhang et al. [15], respectively. For the Indonesian cattle genotypes, 62% of autosomal SNPs remained after cleaning (S2 Table). The results of the population structure analyses are summarized in Table 1. Variability of the samples in each population was proven by the negative off-diagonal variances in the GRM analysis. Although BALI and ONG appeared to be less variable compared with the others, these samples were used as they were obtained from three different sources. The results of the observed and expected heterozygosity estimates were different for each breed. BALI had low heterozygosity compared with other cattle breeds (i.e., 0.12 and 0.08 for the observed and expected heterozygosity, respectively). Among Indonesian cattle, ONG had the highest observed and expected heterozygosity (i.e., 0.31 and 0.25, respectively). The results also indicated an order of heterozygosity as follows: *Bos taurus* > *Bos indicus* > *Bos javanicus*. The inbreeding coefficient (*F*_IS_) values were high in Thailand cattle (TH) (i.e., -0.15). BALI, MAD, BRE and ONG had the same inbreeding index (-0.16), which was slightly higher than that of KBO and ACE (-0.17), followed by NEL (-0.20) and taurines (Simmental/SIM and Limousine/LM; -0.24), while PES had the lowest (-0.27). However, the *F*_IS_ of all the cattle observed in this study were negative, which indicated that the cattle had low levels of inbreeding.

**Table 1:** Data summary for observed cattle populations.

Breed	No. Samples	Observed SNPs in BTA	Cleaned SNPs in BTA	*H*_*o*_^1^	*H*_*e*_^2^	*F*_IS_^3^	GRM^4^		Adjacent LD^5^ (SD)	Recent *N*_*e*_ ^6^
							Diagonal	Off-diagonal		
Bali	54	52,886	32,847	0.12	0.08	-0.16	0.57	-0.011	0.39 (0.40)	96
Madura	37	52,886	32,847	0.26	0.21	-0.16	0.69	-0.019	0.21 (0.28)	150
Jabres	30	52,886	32,847	0.26	0.21	-0.16	0.67	-0.023	0.18 (0.26)	139
Ongole grade	83	52,886	32,069	0.31	0.25	-0.16	0.85	-0.010	0.15 (0.23)	222
Kebumen ongole	25	52,886	32,847	0.29	0.23	-0.17	0.62	-0.026	0.18 (0.26)	117
Aceh	12	42,885	23,598	0.25	0.19	-0.17	0.50	-0.046	0.25 (0.30)	60
Pesisir	6	42,885	23,598	0.24	0.18	-0.27	0.31	-0.063	0.52 (0.38)	15
Thailand	23	38,650	24,667	0.32	0.26	-0.15	0.68	-0.031	0.19 (0.27)	83
Nelore	20	42,885	23,598	0.24	0.18	-0.20	0.57	-0.030	0.20 (0.27)	66
Limousine	20	42,885	23,598	0.40	0.30	-0.24	0.85	-0.045	0.19 (0.26)	93
Simmental	20	42,885	23,598	0.40	0.30	-0.24	0.84	-0.044	0.20 (0.27)	85

^1^ Observed heterozygosity.

^2^ Expected heterozygosity.

^3^ Inbreeding coefficients.

^4^ Average of the genomic relationship matrix referring to the inbreeding of the animal itself (Diagonal) and referring to relationship between animals in the population (Off-diagonal).

^5^ Linkage disequilibrium estimated by the *r*^*2*^ method.

^6^ Effective population size.

Population differentiation based on the *F*_ST_ values accounts for the discrepancy within Indonesian cattle populations, as well as for their relatedness levels with other indicine and taurine cattle (S3 Table; lower diagonal). BALI are an out-group population among Indonesian cattle; most of the other Indonesian cattle populations tend to be closely related (*F*_ST_ ≤ 7.36 × 10^−3)^, whereas BALI has an *F*_ST_ value > 0.02 for its relationship with Indonesian cattle. MAD and BRE had the closest relationship. Indonesian cattle, except for BALI, were closer to TH and NEL and very distant from LM and SIM. This relationship pattern was confirmed by the results of Nei’s genetic distance analysis (S3 Table; upper diagonal). These results indicate that almost all Indonesian cattle (except BALI) tend to be referred to as the indicine type.

**Fig 1:**
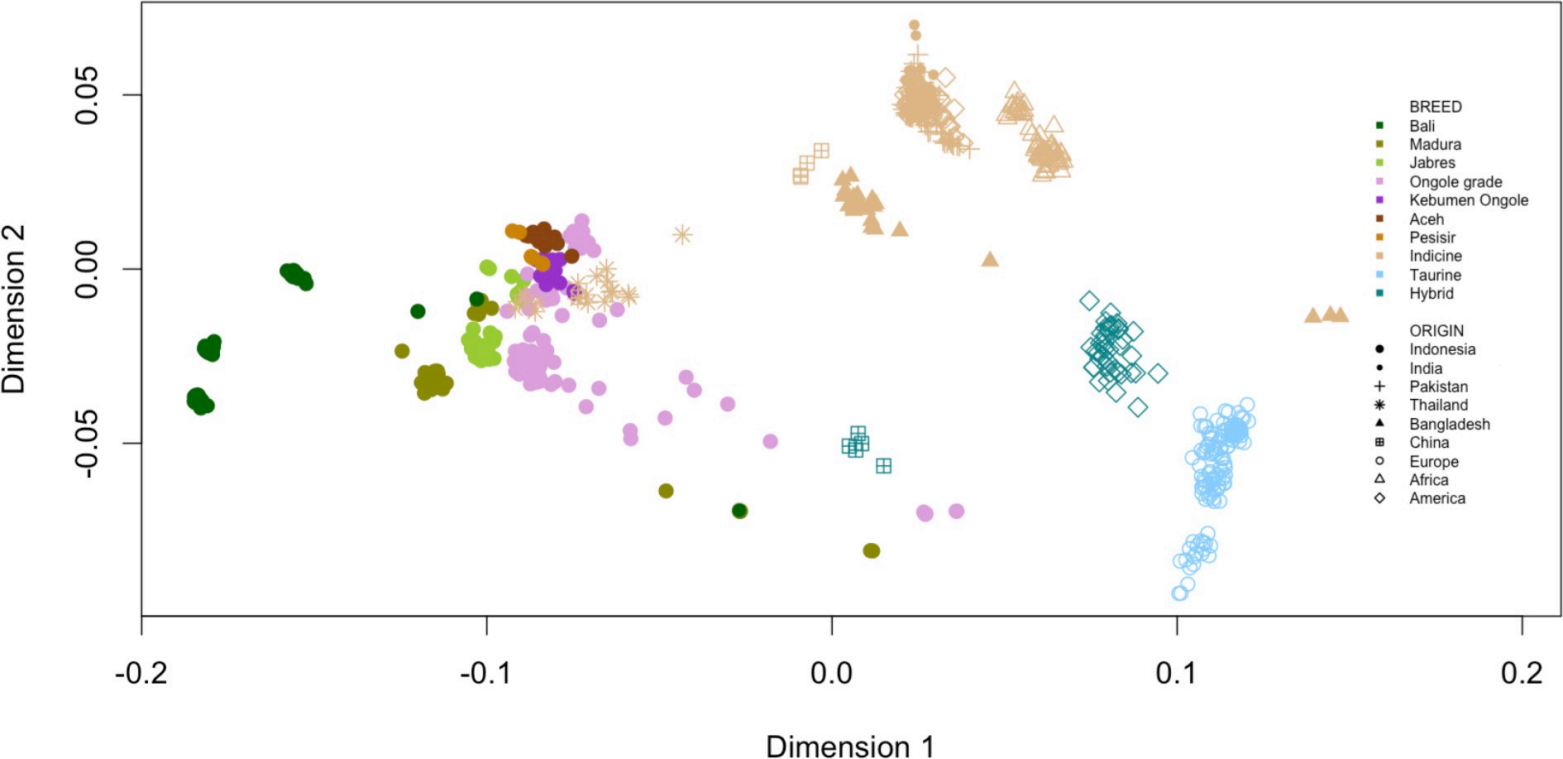
The multi-dimensional scaling plot of Indonesian cattle breeds in the worldwide population context. Points were characterized based on color and shape to define the cattle breed and its origin, respectively.

The MDS results are shown in Fig 1. All samples listed in S1 Table were used to describe the coordinate positions of Indonesian cattle in a worldwide population context. The Indonesian cattle are indicated by the different-colored bold circles, whereas other indicines and taurines are indicated by the different light brown and light blue shapes, respectively. Since African taurine samples were not included, our MDS plot could be considered as a magnified version of the previous principal component analysis performed by Decker et al. [4]. This resulted in a resolution which enabled estimation of the genetic diversity within Indonesian cattle breeds and assessment of their relatedness to populations worldwide. Interestingly, BALI are located at one corner of the triangular shape within the main indicine and taurine cluster, whereas the other Indonesian cattle are located inside of that triangle and forming layers with the following order from the BALI corner: MAD, BRE and then a cluster of ONG, KBO, ACE and PES. TH appear after the ONG and tend to be located towards the main indicine corner. Some of the ONG and MAD are also scattered across the taurine corner.

### Population ancestries and migration events

**Fig 2:**
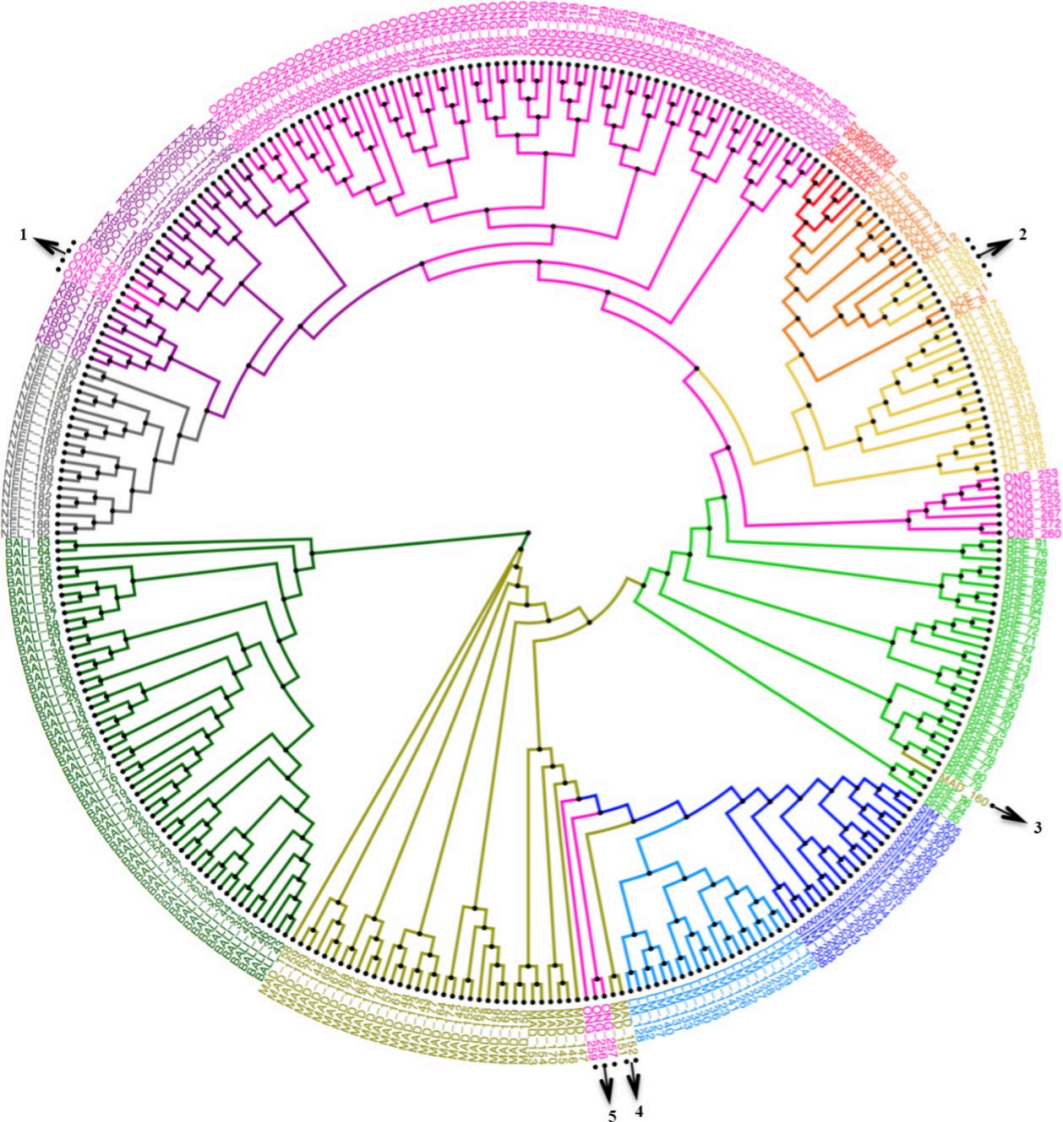
Phylogeny of Indonesian cattle breeds in related with Nelore, zebu in Thailand, and representative taurines (Simmental and Limousine). The phylogeny was rooted in Bali cattle (domesticated *Bos javanicus*). Colors were used to define the cattle breeds, i.e. dark green for Bali, olive green for Madura, chartreuse for Jabres, pink for Ongole grade, purple for Kebumen ongole, red for Pesisir, orange for Aceh, yellow for zebu in Thailand, dark grey for Nelore, dark blue for Simmental, and light blue for Limousine. Individual outliers that respect to their breeds were marked with numbered arrows (1 to 5) and described in the Results part.

**Fig 3:**
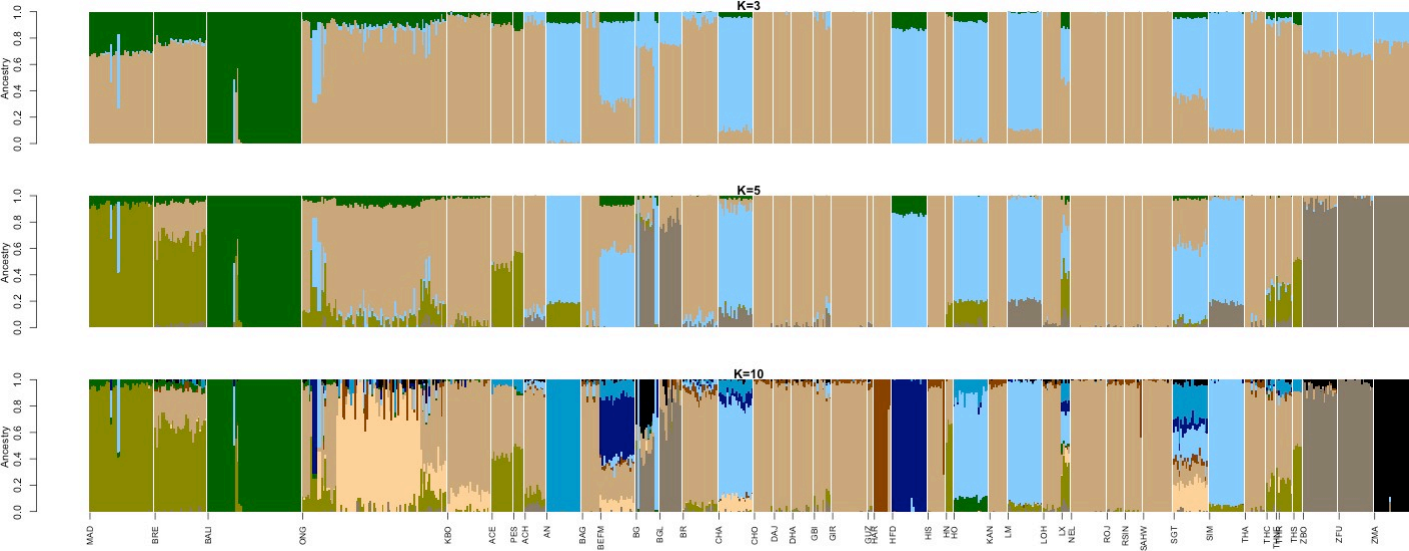
Ancestry models for Indonesian cattle in the worldwide population context. Each vertical line represents one individual. Breed names were abbreviated as in S1 Table. Every K is model referred to the number of estimated ancestors, which were defined by the colors. Model was started from K = 3 and defined *Bos javanicus* (dark green), indicine (light brown), and taurine (light blue) ancestries. K = 10 was a sensible modeling choice based on error cross-validation analysis.

**Fig 4:**
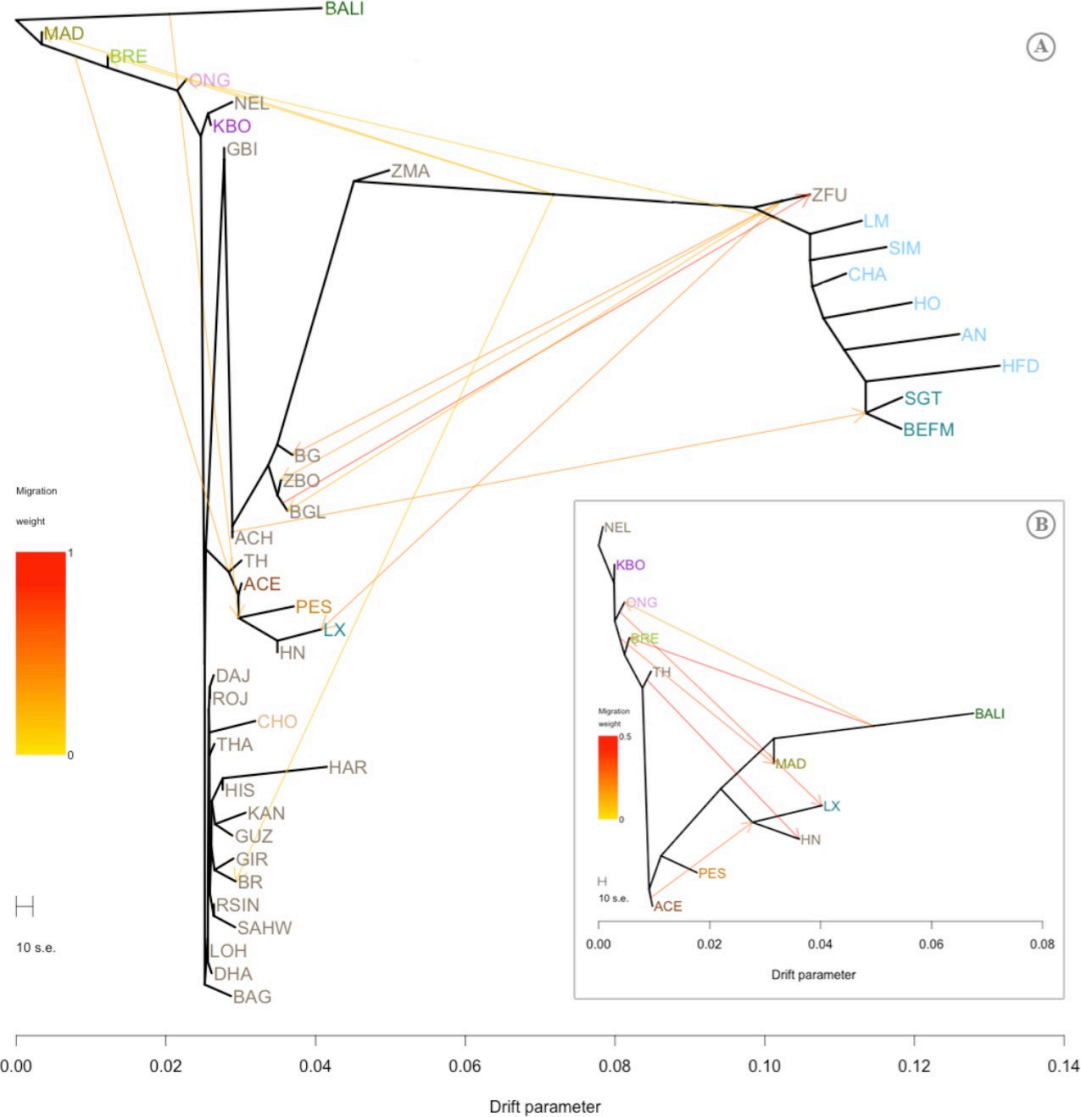
Inferred cattle trees with migration events. Tree models (A, B) were generated based on the two adjusted roots, i.e. Bali (BALI) and Nelore (NEL) cattle, respectively. Breed names were abbreviated as in S1 Table. Worldwide cattle were colored according to their breed types, i.e. grey for indicine, light blue for taurine, and dark blue for hybrid, while colors for Indonesian cattle used to note their population divergences. Migration edges were marked as arrows in the direction from migrant’s origin to the recipient breed and heat-colored according to the mixtures percent.

The possible ancestries of the Indonesian cattle are illustrated using phylogenetic trees (Fig 2), admixture (Fig 3) and TreeMix (Fig 4). Similar initial results were observed from the phylogeny and TreeMix analyses. MAD cattle were directly linked to BALI, as well as KBO which were closely linked to NEL, while ONG and BRE were positioned between KBO and MAD. ACE and PES were clustered with TH and tended to form another group of indicines. In the phylogeny trees, several individuals appeared as outliers and were positioned in different population clades. These individuals are indicated by arrows and are numbered from 1 to 5. Individual outliers 1 and 3 are possibly cattle in the population that are closely related to the next population (i.e., the relationships between ONG and KBO and between MAD and BRE for outliers 1 and 3, respectively). Outlier 2 was the TH that originated from the southern region of Thailand [15], close to Aceh, a region where ACE exist. Crosses between Indonesian cattle (ONG and MAD) and taurine (LM and SIM) were identified as outliers 4 and 5 and this was proven by the admixture and TreeMix analyses shown below.

The results of the admixture analysis clearly described the genetic structure of Indonesian cattle populations by the ancestry-based model in the context of worldwide populations (Fig 3). We performed admixture analysis with K = 2–25; K = 10 resulted in the minimum number of errors after cross validation (i.e., 0.41418) (S2 Fig). The admixture result at K = 3 clearly shows *Bos javanicus* (BALI), *Bos indicus* and *Bos taurus* mixtures in the cattle populations. Indicine was dominant in the structure of Indonesian cattle, especially in KBO, which is 97% indicine on average. Using K = 10, we identified an independent mixture in several ONG individuals that originated from the Beef Cattle Research Station (BCRS) in Indonesia [13], which is similar to the structure of Beef Master (BEFM) and Santa Gertrudis (SGT). Because ONG was the indicine originally used to create SGT and BEFM [13], we estimated that the dominance of ONG in BCRS represents another type of ONG mixture. A dominant and independent mixture of MAD, likely related to BALI, was also identified in the structure of other Indonesian, Thai and Chinese cattle. This finding is consistent with data from a previous study conducted by Decker et al. [4]. Furthermore, small proportions of taurine mixtures were also identified in Indonesian cattle.

Several migration events of Indonesian cattle populations were revealed by the TreeMix analysis (Fig 4). Migration edges that best fit the data were selected if they had positive values, as shown in S3 and S4 Figs by the basal color. Clear migration edges started from BALI and headed toward ONG, BRE, and the branch of PES and Chinese cattle (Hainan / HN and Luxi / LX). The edge from MAD also headed toward a branch of ACE and TH. Since BALI and MAD were directly related in the phylogeny and TreeMix trees, the migration lines emanating from them were presumed to be the *Bos javanicus* introgression. Furthermore, ONG, ACE, and TH had a corresponding impact on Chinese cattle, with a distinctly visible migration event. The directions of these migrations correspond to the introgression of the hybridization of *Bos indicus* and *Bos javanicus* into Chinese cattle. When the root was placed in NEL (Fig 4B), the branch between ONG and BRE also had a migration edge toward MAD, indicating an introgression of *Bos indicus* ancestry in the formation of MAD cattle. Again, an introgression of taurine into Indonesian cattle was identified, with the migration edge leading from the taurine branch to ONG cattle.

We generated the *f3* statistic to gain a better understanding of the possible ancestral mixtures in Indonesian cattle. Table 2 lists the most significant *f3* statistics. BALI introgressions were identified in MAD, BRE, and ONG. These introgression patterns in MAD and BRE were previously identified [4], as was the introgression of BALI in ONG [13]. However, we identified MAD introgression in BRE with a lower Z-score compared with the *f3* statistic of the BALI introgression. This indicates that MAD had a role in the introgression of *Bos javanicus* in BRE. Furthermore, indicine introgression patterns in MAD, BRE, and ONG could be differentiated. Indicine populations based on MAD may have been related to ACE, whereas indicine populations based on ONG and BRE may have been related to NEL.

**Table 2:** The most significant *f3* statistics showing the possible ancestors mixtures in Jabres, Madura, and Ongole grade cattle population.

Population A	Population B	Population C	*f3* statistic	Standard Error	Z-score
Jabres	Nelore	Bali	-0.00967	0.00043	-22.24
Jabres	Bali	Kebumen ongole	-0.00906	0.00039	-23.09
Jabres	Bali	Thailand	-0.00750	0.00037	-20.34
Jabres	Aceh	Bali	-0.00748	0.00038	-19.92
Jabres	Bali	Pesisir	-0.00483	0.00047	-10.26
Jabres	Bali	Ongole grade	-0.00424	0.00036	-11.77
Jabres	Nelore	Madura	-0.00336	0.00009	-36.18
Jabres	Madura	Kebumen ongole	-0.00319	0.00011	-30.12
Jabres	Madura	Thailand	-0.00180	0.00010	-18.55
Jabres	Ongole grade	Madura	-0.00178	0.00008	-22.57
Madura	Nelore	Bali	-0.00760	0.00049	-15.41
Madura	Aceh	Bali	-0.00751	0.00041	-18.24
Madura	Bali	Kebumen ongole	-0.00716	0.00045	-15.83
Madura	Bali	Thailand	-0.00699	0.00042	-16.64
Madura	Bali	Pesisir	-0.00539	0.00047	-11.45
Ongole grade	Nelore	Bali	-0.00263	0.00023	-11.45
Ongole grade	Bali	Kebumen ongole	-0.00176	0.00024	-7.43

### Demographic trends

**Fig 5:**
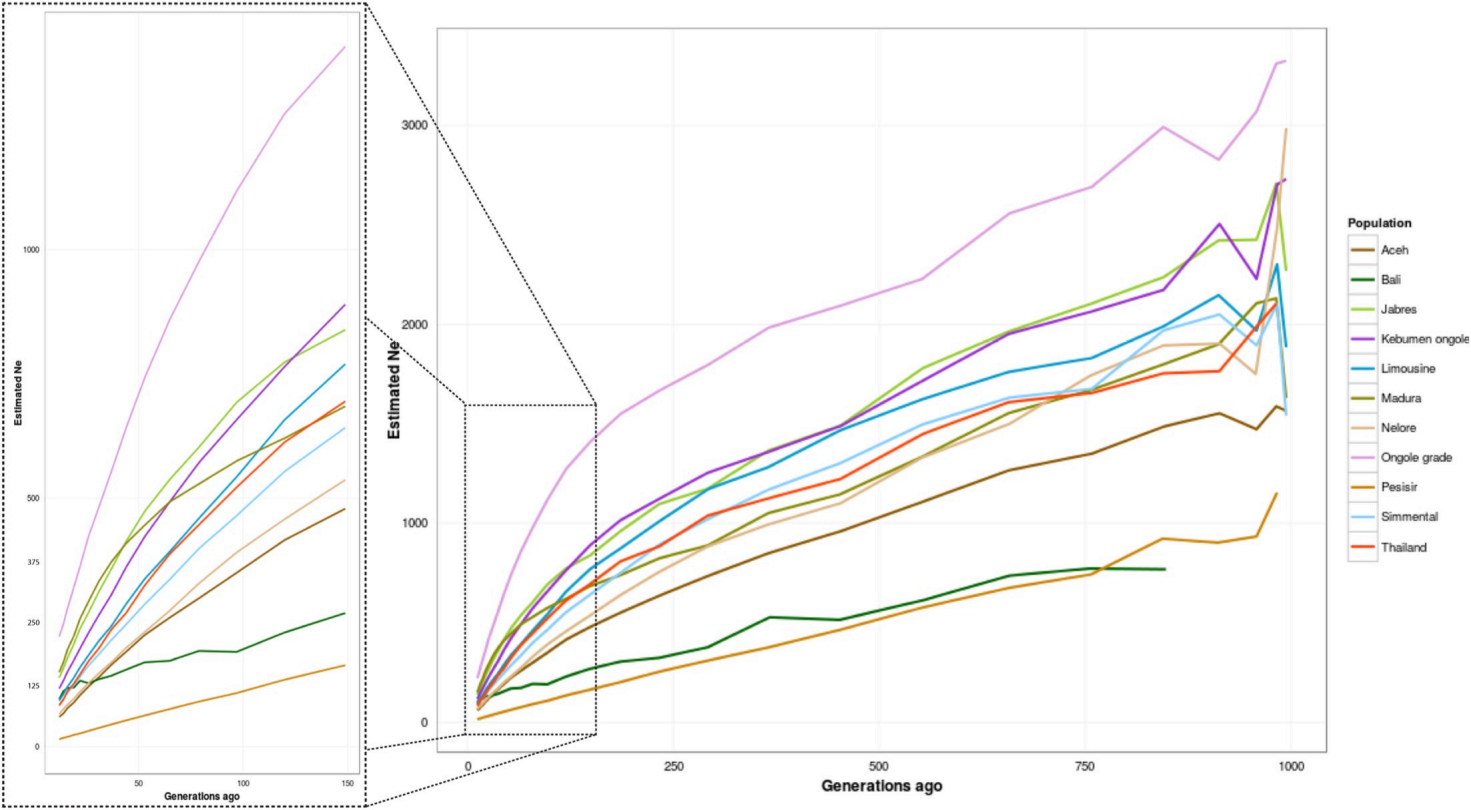
Historical trends of the effective population size for Indonesian cattle, indicines (Thailand cattle, Nelore), and taurines (Simmental, Limousine). Trends of the effective population size were set to the times in ~12 to ~994 generations ago. Lines were colored according to the breeds. Figure was zoomed for the 12 to 150 generations ago.

The historical *N*_*e*_ was estimated based on the value of LD across the genome and was used as a representation of demographic changes in cattle populations. The adjacent LD and recent *N*_*e*_ values of the observed cattle breeds (all Indonesian cattle, TH, NEL, LM, and SIM) are summarized in Table 1 and the averages, based on genetic distances, are shown in S4 Table. Finally, distance ranges and the *N*_*e*_ over ~1,000 generations are shown in Fig 5. The sample numbers of BALI, MAD, BRE, ONG and KBO were sufficiently large to be used in the LD analysis of the cattle [39]. However, because the sample numbers of ACE and PES were very limited, their LD and *N*_*e*_ analyses results, and those of other foreign breeds, should be considered as rough approximations only.

Among Indonesian cattle, a low level of LD was identified for ONG (0.15), followed by KBO and BRE (0.18), MAD (0.21), ACE (0.25), BALI (0.39) and PES (0.52). The foreign TH had a similar LD value as LM (i.e., 0.19) and NEL also had a similar LD value as SIM (i.e. 0.20). The highest *N*_*e*_ value for 12 generations ago was found for ONG (222), followed by these populations in descending order: MAD (150), BRE (139), KBO (117), BALI (96), LM (93), SIM (85), TH (83), NEL (66), ACE (60) and PES (15). The *N*_*e*_ value of all cattle breeds showed a declining trend from the past to the present. However, BALI and MAD showed more interesting trends: an increase in the *N*_*e*_ value at 22, 79, and 367 generations ago for BALI and a small decline in *N*_*e*_ for MAD at 97 to 44 generations ago. Furthermore, the *N*_*e*_ trend for BALI can be traced back to only ~848 generations ago, which is shorter than the other breeds. The maximum generation revealed by the *N*_*e*_ analysis may also indicate the time of domestication of the population.

## Discussion

Most recent studies of cattle genetic differentiation analyze the population structure, trace the ancestral admixtures and predict the demographic history. In this study, we performed these analyses using genome-wide SNP data for Indonesian cattle to advance our knowledge about their diversity in a worldwide population context. *Bos javanicus* originated from Indonesia, and BALI were domesticated from that species [9, 14, 40]. Previous studies also identified a massive introgression of *Bos indicus* into the Indonesian cattle population [9, 13, 14, 41]. In this study, we looked for evidence of a relationship between *Bos javanicus* and *Bos indicus* in seven Indonesian cattle populations.

BALI have a unique population structure; low heterozygosity in this population does not mean they have a lower sample variability or a higher inbreeding level compared with other Indonesian cattle. In our analysis, their sample variability and inbreeding level were equal to those of ONG, which had the highest heterozygosity among Indonesian cattle (Table 1). Such typical patterns of estimated heterozygosity for Indonesian cattle were also identified previously [9, 13]. Since we used three sources of BALI populations, the structure of each of those local populations may have influenced our results. Mohamad et al. [9] assumed that inbreeding within the local populations might affect the population structure to some extent. We suggest that the low heterozygosity in BALI might be caused by their common parentage due to decades of isolation and the restricted use of foreign bulls in Bali [41]. BALI in other parts of Indonesia were also housed separately from zebu [9]. Therefore, only a low level of admixture from other breeds was present in BALI, as also identified in other studies [13, 14]. Furthermore, the high heterozygosity in ONG was influenced by the use of different bulls in mating programs.

Despite most Indonesian cattle in the genetic distance analyses being identified as indicine type (S3 Table) [9], the layers in the MDS plot that spanned between *Bos javanicus* and *Bos indicus* clearly indicated each Indonesian cattle population and explained the formation of several hybrid Indonesian cattle of *Bos javanicus* and *Bos indicus* (Fig 1). The result of the admixture analysis (Fig 3) supported this idea. In general, there were three major types of Indonesian cattle: populations with pure *Bos javanicus* ancestry (BALI), populations with *Bos indicus* dominating (KBO) and populations with hybrid characteristics (MAD, BRE, ONG, ACE, and PES). ONG from BCRS have a distinct admixture, but one in which *Bos indicus* dominates. This distinct mixture of ONG might indicate the pure ONG breed, as cattle from BCRS were selected to establish a breeding stock of ONG with superior characteristics and high purity [42].

Interestingly, KBO were directly linked to NEL in the trees (Figs 2 and 4), in which NEL were Ongole cattle imported from India to Brazil at the end of the 18^th^ century [2, 6]. Presumably, KBO and NEL have a close ancestral relationship, as, during the Dutch colonial period, Ongole bulls were imported to Indonesia from Nellore province, India, which was also around the time they were imported to Brazil [2, 6, 13]. In Indonesia, those imported Ongole were initially kept on Sumbawa Island before being distributed to Java and nationally [13, 40]. At that time, they were used as draft animals on paved roads [6]. We hypothesized that KBO are Ongole cattle, as they were raised in the region where the first and longest paved roads were built in Java [43]. Moreover, we suggest that the importation of Ongole cattle to Indonesia comprised both admixed and purebred animals, such as ONG and KBO, respectively. This is supported by the results of the *f3* statistic, which indicates that both NEL and KBO were identified as possible ancestors of ONG (Table 2). However, since KBO were usually maintained without a specific breeding program that would have ensured their purity, we recently identified low levels of admixed in KBO (< 3%), indicating *Bos javanicus* and *Bos taurus* ancestries. These mixtures are most likely due to crosses between KBO and ONG or other taurine breeds owned by the farmers. Local farmers often termed the KBO as “Madras” cattle, which was the former name of Chennai city, India. This might be indicative of the place of origin of the KBO cattle that were raised in that area. The regions of Ongole, Nellore, and Madras are similarly located along the southeastern coast of India, where the Ongole cattle breed originated [13, 44, 45].

The presence of ACE and PES on the node close to TH is another indication of the migration of cattle into Indonesia (Fig 2). Similar results were also observed in a previous study of the genetic diversity of Thai cattle, suggesting another possible origin of the *Bos indicus* ancestor [15]. Two Bangladeshi cattle (BG and BGL) used in the population migration study did not correspond to Indonesian and TH (Fig 3). Bangladeshi cattle were previously identified in a distinctive cluster with other Asian indicines [46]. This supports the hypothesis that the possible migration of indicines to Indonesia might have been due to their direct introduction by humans via sea routes. The mountains on the border between Bangladesh and Myanmar likely prevented cattle migration [46]. Based on all these results, we estimated that *Bos indicus* were imported in the 9^th^ and 10^th^ centuries (during the Bronze Age), as historians have suggested that people travelled by sea from India to Southeast Asia and China, or in the opposite direction, for trade and religious purposes. Indeed, archaeological evidence of similar characteristics in sculptures, as well as other ancient goods, have been found in these regions [8, 10, 47]. During this period, indicines likely contributed to the formation of MAD, as the most significant *f3* statistics indicate that the possible ancestors of MAD were ACE and BALI (Table 2). The Sinhalese, who were also a seafaring nation at that time, may also have transported indicine cattle to Indonesia [9, 48].

Alongside BALI, MAD also contributed their *Bos javanicus* ancestry to other breeds. This was seen as an independent mixture of MAD that was present in other breeds (Fig 3), as well as the migration edges of BALI and MAD into BRE, ONG, ACE, and PES. Furthermore, based on the result of the *f3* statistical analysis, MAD were identified as the possible ancestors of BRE (Table 2). The introgression of *Bos javanicus* was also identified in cattle from Thailand and China [4, 15].

The demographic history of populations can be inferred based on the LD and *N*_*e*_ values [18]. In our analysis, ONG with the lowest LD had the highest *N*_*e*_ value. In contrast, PES with the highest LD had the lowest *N*_*e*_ value (Table 1). It was expected that populations with low LD would have a high *N*_*e*_ and *vice versa*. However, the value of LD varies depending on the population’s demographic history [36] and *N*_*e*_ varies depending on the applied selection and breeding programs in the population [49]. LD and *N*_*e*_ values will change if events, such as genetic drift, migration, selection, mutation and changes in the recombination rate, occur in the population [18, 36]. Hence, BALI with a high LD had a mid-range level of *N*_*e*_ due to prior increases in the *N*_*e*_ value in past generations (Fig 5; S4 Table). Assuming that a breeding generation in cattle is ~4 years [18], 22 generations ago, when the recent *N*_*e*_ value increased in BALI, could have been the period when BALI were distributed to other regions in Indonesia, which might have led to the increase in their population numbers and increased genetic variation [10, 41]. Our results also supported the hypothesis that the domestication of BALI occurred later than that of other breeds [6], but earlier than the previous estimate of ~1500 AD, as predicted by Sutarno and Setyawan [10]. We suggest that BALI were domesticated ~3,400 years ago. Moreover, there was a slow reduction in the *N*_*e*_ value of MAD cattle from 97 to 44 generations ago (Fig 5), which might indicate an effort to maintain their genetic variation, probably due to the development, distribution, and crosses of MAD with other breeds [10]. However, at the beginning of the 20^th^ century, their *N*_*e*_ value dropped sharply, concomitant with a restriction of the use of other breeds in Madura Island [41]. Interestingly, recent *N*_*e*_ values for the majority of Indonesian cattle are still higher than the limit assigned by The Food and Agricultural Organization of the United Nations (i.e., 50 per generation) to maintain breed fitness [50]. However, the declining trend in the *N*_*e*_ value is of concern; therefore, specific breeding programs are urgently needed to enable inbreeding control for these Indonesian cattle.

## Conclusion

We identified genome-wide evidence of Indonesian cattle diversity in this study based on three distinct cattle types (i.e., cattle of *Bos javanicus*, *Bos indicus*, and their hybrids) to gain a better understanding of their origins and establishment in Indonesia. Additionally, we investigated thoroughbred Ongole cattle that have been raised by farmers in Kebumen Regency and determined that efforts are needed to maintain their genetic purity. Furthermore, improvement of the genetic variability of cattle populations with a low effective population size is also needed to maintain their sustainability.

## Supporting information

S1 Fig Image of Indonesian cattle breeds. (TIF)

S2 Fig Cross-validation plot of the admixture analysis. (PDF)

S3 Fig Plot of residuals from TreeMix analysis depicted in Fig 4A when tree was rooted in Bali cattle. (TIFF)

S4 Fig Plot of residuals from TreeMix analysis depicted in Fig 4B when tree was rooted in Nelore cattle. (TIFF)

S1 Table Samples used in the analysis. (DOCX)

S2 Table Detail numbers of removed data due to cleaning process. (DOCX)

S3 Table Pairwise *F*_ST_ (lower diagonal) and Nei’s genetic distances between populations (upper diagonal). (DOCX)

S4 Table The historical effective population size (*N*_*e*_). (DOCX)

S1 File (TXT)
