# Supplementary figures and images for: An insight into the evolutionary history of Indonesian cattle assessed by whole genome data analysis
Source: PLoS One. 2020 Nov 10;15(11):e0241038. doi: 10.1371/journal.pone.0241038 (PMC7654832; doi:10.1371/journal.pone.0241038)

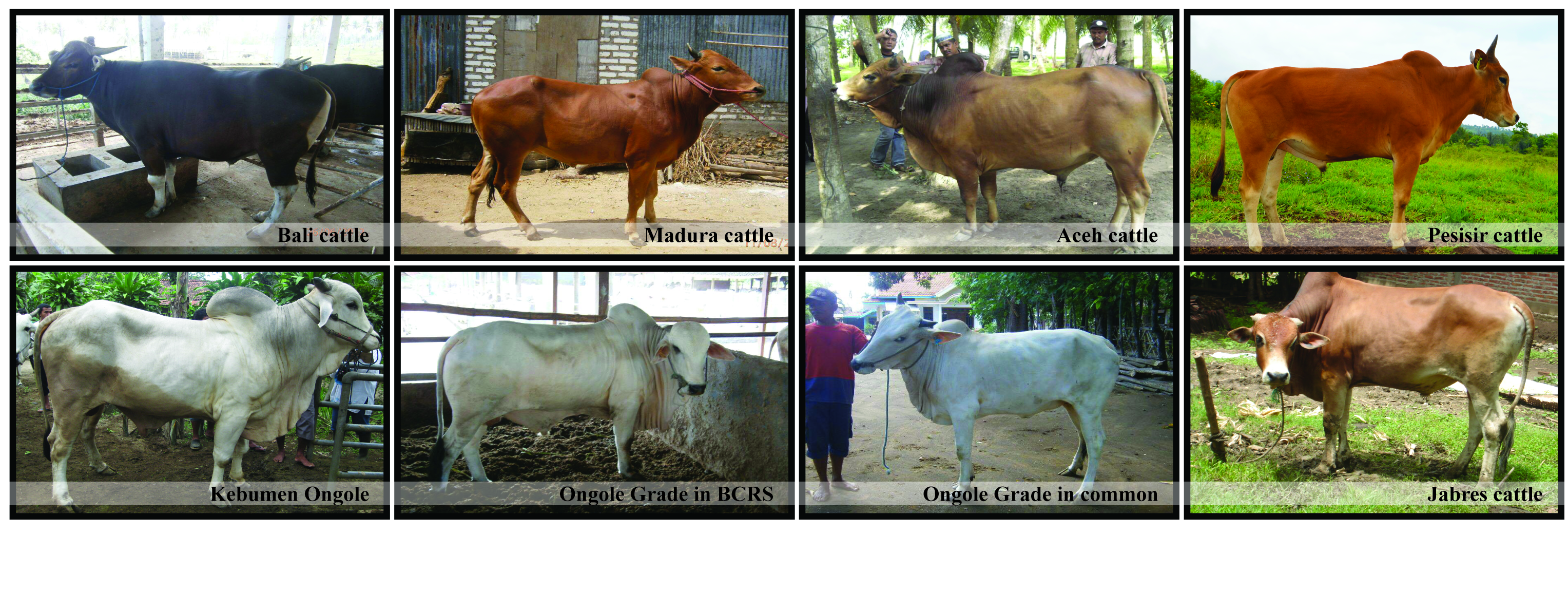

Supplement: S1 Fig — (TIF) [file pone.0241038.s001.tif]

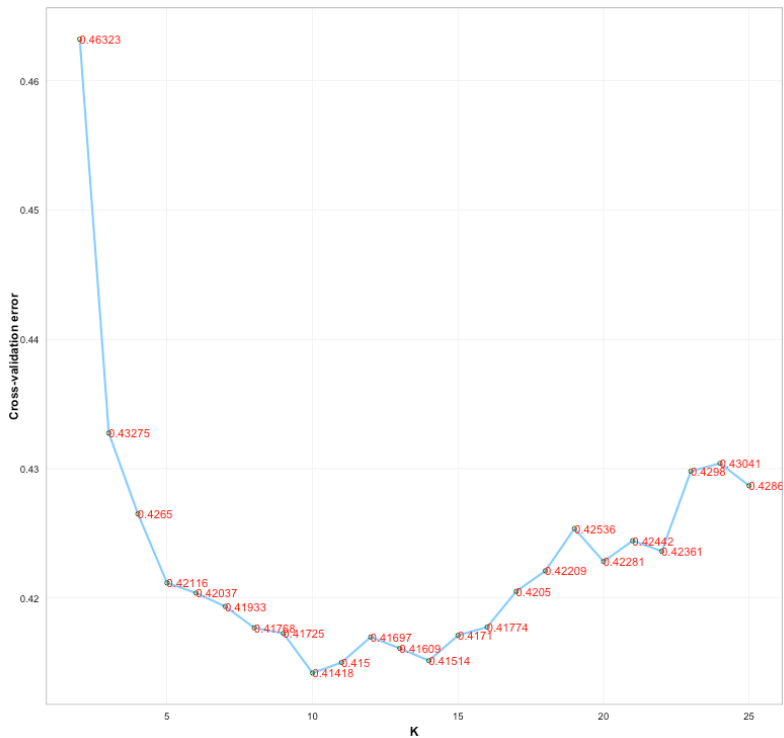

Supplement: S2 Fig — (PDF) [file pone.0241038.s002.pdf]

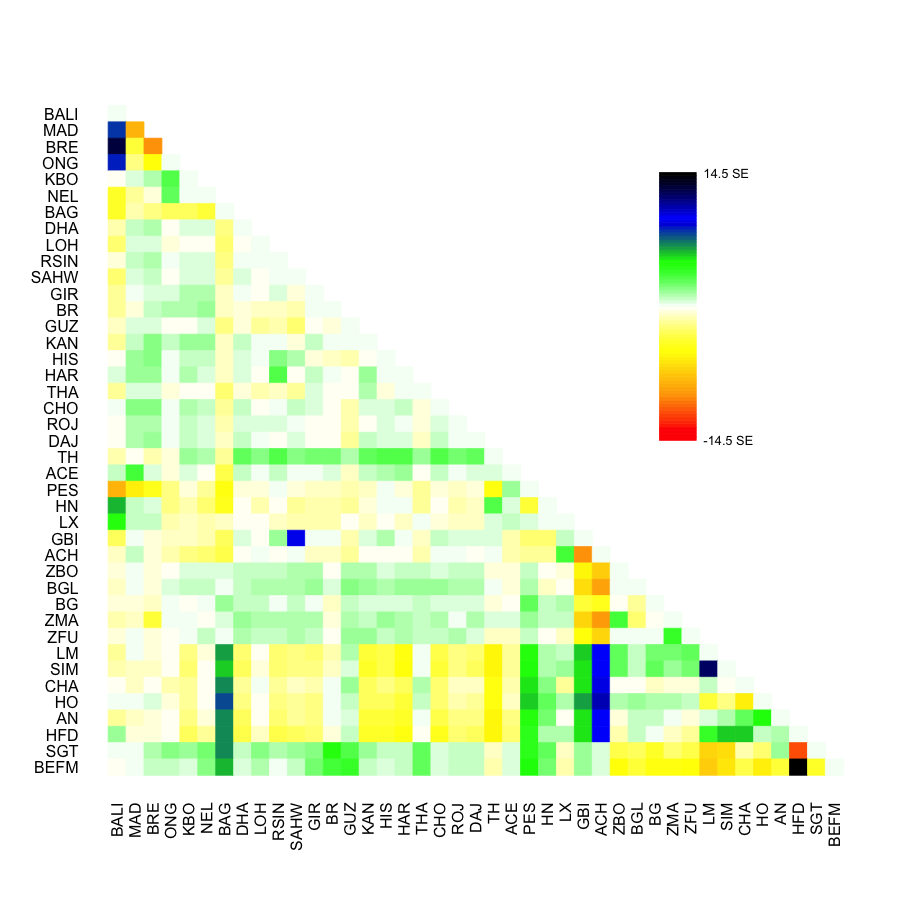

Supplement: S3 Fig — (TIFF) [file pone.0241038.s003.tiff]

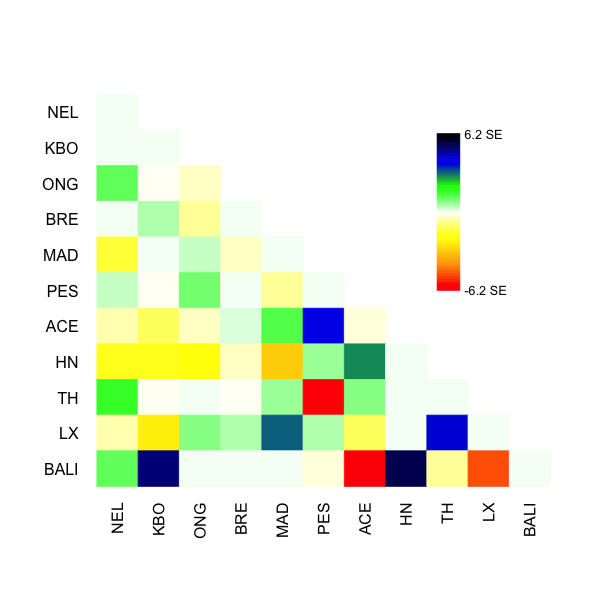

Supplement: S4 Fig — (TIFF) [file pone.0241038.s004.tiff]
