# Supplementary material for: An insight into the evolutionary history of Indonesian cattle assessed by whole genome data analysis
Source: PLoS One. 2020 Nov 10;15(11):e0241038. doi: 10.1371/journal.pone.0241038 (PMC7654832; doi:10.1371/journal.pone.0241038)
Supplement: S1 Table — (DOCX) [file pone.0241038.s005.docx]

**S1 Table** Samples used in the analysis.

| **Breed** | **Taxa** | **Abbreviation** | **No. samples** | **No. samples from other sources^1^** | **Origin** |
| --- | --- | --- | --- | --- | --- |
| Bali | *Bos javanicus* | BALI | 54 | 20(a), 18(b) | Indonesia |
| Madura | *Bos indicus* | MAD | 37 | 7(a) | Indonesia |
| Jabres | *Bos indicus* | BRE | 30 | 9(a) | Indonesia |
| Ongole Grade | *Bos indicus* | ONG | 83 | 20(a), 48(b) | Indonesia |
| Kebumen Ongole | *Bos indicus* | KBO | 25 |  | Indonesia |
| Aceh | *Bos indicus* | ACE | 12 | 12(a) | Indonesia |
| Pesisir | *Bos indicus* | PES | 6 | 6(a) | Indonesia |
| Kho-Khaolumpoon | *Bos indicus* | THR (TH) | 8 | 8(c) | Thailand |
| Kho-Isaan | *Bos indicus* | THNE (TH) | 5 | 5(c) | Thailand |
| Kho-Lan | *Bos indicus* | THC (TH) | 5 | 5(c) | Thailand |
| Kho-Chon | *Bos indicus* | THS (TH) | 5 | 5(c) | Thailand |
| Bangladeshi zebu 1 | *Bos indicus* | BG | 13 | 13(d) | Bangladesh |
| Bangladeshi zebu 2 | *Bos indicus* | BGL | 14 | 14(d) | Bangladesh |
| Nelore | *Bos indicus* | NEL | 20 | 20(a) | America, imported from India |
| Brahman | *Bos indicus* | BR | 20 | 20(a) | America |
| Achai | *Bos indicus* | ACH | 12 | 12(a) | Pakistan |
| Red Sindhi | *Bos indicus* | RSIN | 10 | 10(a) | Pakistan |
| Cholistani | *Bos indicus* | CHO | 11 | 11(a) | Pakistan |
| Gir | *Bos indicus* | GIR | 20 | 20(a) | India |
| Guzerat | *Bos indicus* | GUZ | 3 | 3(a) | India |
| Hariana | *Bos indicus* | HAR | 10 | 10(a) | India |
| Dajal | *Bos indicus* | DAJ | 10 | 10(a) | Pakistan |
| Bhagnari | *Bos indicus* | BAG | 10 | 10(a) | Pakistan |
| Gabrali | *Bos indicus* | GBI | 10 | 10(a) | Pakistan |
| Kankraj | *Bos indicus* | KAN | 10 | 10(a) | India |
| Lohani | *Bos indicus* | LOH | 10 | 10(a) | Pakistan |
| Dhanni | *Bos indicus* | DHA | 12 | 12(a) | Pakistan |
| Hissar | *Bos indicus* | HIS | 10 | 10(a) | Pakistan |
| Sahiwal | *Bos indicus* | SAHW | 17 | 17(a) | Pakistan |
| Tharparkar | *Bos indicus* | THA | 12 | 12(a) | Pakistan |
| Rojhan | *Bos indicus* | ROJ | 10 | 10(a) | Pakistan |
| Zebu Fulani | *Bos indicus* | ZFU | 20 | 20(a) | Africa |
| Zebu Bororo | *Bos indicus* | ZBU | 20 | 20(a) | Africa |
| Madagascar Zebu | *Bos indicus* | ZMA | 20 | 20(a) | Africa |
| Hainan | *Bos indicus* | HN | 4 | 4(a) | China |
| Luxi | Hybrid | LX | 5 | 5(a) | China |
| Beef Master | Hybrid | BEFM | 20 | 20(a) | America |
| Santa Gertrudis | Hybrid | SGT | 20 | 20(a) | America |
| Simmental | *Bos taurus* | SIM | 20 | 20(a) | Europe |
| Limousine | *Bos taurus* | LM | 20 | 20(a) | Europe |
| Charolais | *Bos taurus* | CHA | 20 | 20(a) | Europe |
| Friesian Holstein | *Bos taurus* | HO | 20 | 20(a) | Europe |
| Angus | *Bos taurus* | AN | 20 | 20(a) | Europe |
| Hereford | *Bos taurus* | HFD | 20 | 20(a) | Europe |

^1^ a: Decker et al. (2014); b: Hartati et al. (2015); c: Wangkumhang et al. (2015), d: Uzzaman et al. (2014).
