# Supplementary material for: An insight into the evolutionary history of Indonesian cattle assessed by whole genome data analysis
Source: PLoS One. 2020 Nov 10;15(11):e0241038. doi: 10.1371/journal.pone.0241038 (PMC7654832; doi:10.1371/journal.pone.0241038)
Supplement: S2 Table — (DOCX) [file pone.0241038.s006.docx]

**S2 Table** Detail numbers of removed data due to cleaning process.

| **Breed^1^** | **Observed SNPs in BTA** | **Removed SNPs on each filtering parameter** | | | **Cleaned SNPs in BTA** |
| --- | --- | --- | --- | --- | --- |
|  |  | **HWE^2^** | **Geno^3^** | **MAF^4^** |  |
| BALI | 52,886 | 2,034 | 2,926 | 15,079 | 32,847 |
| MAD | 52,886 | 2,034 | 2,926 | 15,079 | 32,847 |
| BRE | 52,886 | 2,034 | 2,926 | 15,079 | 32,847 |
| ONG | 52,886 | 2,638 | 1,226 | 16,953 | 32,069 |
| KBO | 52,886 | 2,034 | 2,926 | 15,079 | 32,847 |
| ACE | 42,885 | 18,066 | 157 | 1,064 | 23,598 |
| PES | 42,885 | 18,066 | 157 | 1,064 | 23,598 |
| TH | 38,650 | 3 | 115 | 13,865 | 24,667 |
| NEL | 42,885 | 18,066 | 157 | 1,064 | 23,598 |
| LIM | 42,885 | 18,066 | 157 | 1,064 | 23,598 |
| SIM | 42,885 | 18,066 | 157 | 1,064 | 23,598 |

^1^ Abbreviated name of cattle breed based on Table S1.

^2^ Hardy-Weinberg Equilibrium exact test.

^3^ Missing genotypes data.

^4^ Minor allele frequencies threshold.
