# Supplementary material for: An insight into the evolutionary history of Indonesian cattle assessed by whole genome data analysis
Source: PLoS One. 2020 Nov 10;15(11):e0241038. doi: 10.1371/journal.pone.0241038 (PMC7654832; doi:10.1371/journal.pone.0241038)
Supplement: S3 Table — (DOCX) [file pone.0241038.s007.docx]

**S3 Table** Pairwise *F*_ST_ (lower diagonal) and Nei’s genetic distances between populations (upper diagonal).

|  | **BALI** | **MAD** | **BRE** | **ONG** | **KBO** | **PES** | **ACE** | **TH** | **NEL** | **LM** | **SIM** |
| --- | --- | --- | --- | --- | --- | --- | --- | --- | --- | --- | --- |
| **BALI** | 0 | 0.02391 | 0.02669 | 0.03071 | 0.03428 | 0.03759 | 0.03464 | 0.03370 | 0.03481 | 0.05551 | 0.05698 |
| **MAD** | 0.02479 | 0 | 0.00192 | 0.00588 | 0.00607 | 0.01010 | 0.00603 | 0.00626 | 0.00705 | 0.03381 | 0.03565 |
| **BRE** | 0.02761 | 0.00091 | 0 | 0.00424 | 0.00386 | 0.00888 | 0.00450 | 0.00477 | 0.00501 | 0.03416 | 0.03593 |
| **ONG** | 0.03136 | 0.00523 | 0.00346 | 0 | 0.00363 | 0.01059 | 0.00595 | 0.00633 | 0.00532 | 0.03451 | 0.03624 |
| **KBO** | 0.03519 | 0.00497 | 0.00267 | 0.00271 | 0 | 0.00981 | 0.00498 | 0.00546 | 0.00389 | 0.03564 | 0.03746 |
| **PES** | 0.03812 | 0.00693 | 0.00577 | 0.00736 | 0.00648 | 0 | 0.00805 | 0.01029 | 0.01119 | 0.04002 | 0.04175 |
| **ACE** | 0.03553 | 0.00421 | 0.00265 | 0.00423 | 0.00296 | 0.00429 | 0 | 0.00524 | 0.00651 | 0.03671 | 0.03840 |
| **TH** | 0.03470 | 0.00520 | 0.00365 | 0.00542 | 0.00425 | 0.00759 | 0.00350 | 0 | 0.00514 | 0.03565 | 0.03728 |
| **NEL** | 0.03578 | 0.00586 | 0.00377 | 0.00427 | 0.00252 | 0.00830 | 0.00463 | 0.00393 | 0 | 0.03501 | 0.03676 |
| **LM** | 0.05754 | 0.03339 | 0.03360 | 0.03428 | 0.03494 | 0.03518 | 0.03477 | 0.03505 | 0.03421 | 0 | 0.00997 |
| **SIM** | 0.05915 | 0.03537 | 0.03553 | 0.03613 | 0.03694 | 0.03725 | 0.03671 | 0.03687 | 0.03617 | 0.00733 | 0 |
