# Supplementary material for: An insight into the evolutionary history of Indonesian cattle assessed by whole genome data analysis
Source: PLoS One. 2020 Nov 10;15(11):e0241038. doi: 10.1371/journal.pone.0241038 (PMC7654832; doi:10.1371/journal.pone.0241038)
Supplement: S4 Table — (DOCX) [file pone.0241038.s008.docx]

**S4 Table** The historical effective population size (*N_e_*).

| **BALI** | | | | | | | | | | | | | | | | | | | | | | | | | |
| --- | --- | --- | --- | --- | --- | --- | --- | --- | --- | --- | --- | --- | --- | --- | --- | --- | --- | --- | --- | --- | --- | --- | --- | --- | --- |
| **Fragment size** | **5,512** | **5,034** | **4,500** | **3,987** | **3,755** | **3,278** | **2,956** | **2,640** | **2,499** | **2,141** | **1,765** | **1,610** | **1,408** | **1,237** | **1,047** | **862** | **719** | **556** | **471** | **357** | **265** | **194** | **138** | **78** | **-** |
| ***N_e_*** | **96** | **112** | **118** | **119** | **133** | **128** | **136** | **143** | **155** | **170** | **173** | **193** | **191** | **230** | **269** | **305** | **324** | **377** | **528** | **515** | **613** | **737** | **773** | **769** | **-** |
| **Generation** | **12** | **14** | **16** | **19** | **22** | **26** | **31** | **37** | **44** | **53** | **65** | **79** | **97** | **120** | **149** | **186** | **233** | **292** | **367** | **452** | **553** | **658** | **756** | **848** | **-** |
| **MAD** | | | | | | | | | | | | | | | | | | | | | | | | | |
| **Fragment size** | **124K** | **113K** | **103K** | **92K** | **83K** | **75K** | **66K** | **58K** | **51K** | **45K** | **39K** | **34K** | **29K** | **24K** | **20K** | **17K** | **13K** | **11K** | **8,447** | **6,605** | **4,833** | **3,557** | **2,332** | **1,574** | **85** |
| ***N_e_*** | **150** | **170** | **196** | **223** | **258** | **292** | **333** | **372** | **409** | **446** | **493** | **529** | **575** | **621** | **685** | **740** | **824** | **889** | **1,051** | **1,145** | **1,334** | **1,554** | **1,671** | **1,799** | **1,630** |
| **Generation** | **12** | **14** | **16** | **19** | **22** | **26** | **31** | **37** | **44** | **53** | **65** | **79** | **97** | **120** | **149** | **186** | **233** | **292** | **365** | **452** | **552** | **657** | **758** | **845** | **994** |
| **BRE** | | | | | | | | | | | | | | | | | | | | | | | | | |
| **Fragment size** | **142K** | **129K** | **117K** | **106K** | **95K** | **86K** | **76K** | **67K** | **59K** | **52K** | **45K** | **38K** | **33K** | **28K** | **23K** | **19K** | **15K** | **13K** | **9,631** | **7,551** | **5,517** | **4,081** | **2,729** | **1,791** | **99** |
| ***N_e_*** | **139** | **158** | **179** | **207** | **238** | **270** | **312** | **358** | **413** | **474** | **538** | **603** | **693** | **774** | **839** | **961** | **1,097** | **1,174** | **1,365** | **1,491** | **1,780** | **1,964** | **2,105** | **2,237** | **2,269** |
| **Generation** | **12** | **14** | **16** | **19** | **22** | **26** | **31** | **37** | **44** | **53** | **65** | **79** | **97** | **120** | **149** | **186** | **233** | **292** | **365** | **453** | **553** | **657** | **758** | **845** | **994** |
| **ONG** | | | | | | | | | | | | | | | | | | | | | | | | | |
| **Fragment size** | **164K** | **149K** | **135K** | **122K** | **110K** | **98K** | **87K** | **77K** | **68K** | **59K** | **52K** | **44K** | **38K** | **32K** | **26K** | **22K** | **18K** | **14K** | **11K** | **8,567** | **6,445** | **4,578** | **3,141** | **2,051** | **113** |
| ***N_e_*** | **222** | **247** | **279** | **321** | **363** | **423** | **482** | **554** | **641** | **743** | **860** | **977** | **1,118** | **1,274** | **1,409** | **1,550** | **1,667** | **1,796** | **1,984** | **2,094** | **2,228** | **2,558** | **2,691** | **2,992** | **3,325** |
| **Generation** | **12** | **14** | **16** | **19** | **22** | **26** | **31** | **37** | **44** | **53** | **65** | **79** | **97** | **120** | **149** | **186** | **233** | **292** | **366** | **453** | **552** | **658** | **758** | **845** | **994** |
| **KBO** | | | | | | | | | | | | | | | | | | | | | | | | | |
| **Fragment size** | **122K** | **111K** | **102K** | **91K** | **83K** | **74K** | **66K** | **58K** | **51K** | **45K** | **39K** | **33K** | **29K** | **24K** | **20K** | **17K** | **14K** | **11K** | **8,480** | **6,566** | **4,903** | **3,580** | **2,430** | **1,591** | **88** |
| ***N_e_*** | **117** | **133** | **151** | **174** | **198** | **229** | **265** | **306** | **361** | **423** | **493** | **572** | **658** | **765** | **890** | **1,016** | **1,123** | **1,254** | **1,360** | **1,489** | **1,715** | **1,952** | **2,065** | **2,173** | **2,731** |
| **Generation** | **12** | **14** | **16** | **19** | **22** | **26** | **31** | **37** | **44** | **53** | **65** | **79** | **97** | **120** | **149** | **186** | **233** | **292** | **366** | **453** | **552** | **657** | **758** | **845** | **994** |
| **ACE** | | | | | | | | | | | | | | | | | | | | | | | | | |
| **Fragment size** | **79K** | **73K** | **66K** | **59K** | **54K** | **48K** | **43K** | **38K** | **33K** | **29K** | **26K** | **22K** | **19K** | **16K** | **13K** | **11K** | **9,017** | **7,240** | **5,621** | **4,401** | **3,283** | **2,361** | **1,593** | **1,061** | **60** |
| ***N_e_*** | **60** | **67** | **78** | **89** | **104** | **121** | **140** | **165** | **191** | **225** | **260** | **299** | **350** | **416** | **479** | **551** | **636** | **735** | **849** | **960** | **1,107** | **1,266** | **1,350** | **1,487** | **1,566** |
| **Generation** | **12** | **14** | **16** | **19** | **22** | **26** | **31** | **37** | **44** | **53** | **65** | **79** | **97** | **120** | **149** | **186** | **233** | **292** | **365** | **453** | **552** | **657** | **758** | **846** | **994** |
| **PES** | | | | | | | | | | | | | | | | | | | | | | | | | |
| **Fragment size** | **55K** | **50K** | **45K** | **41K** | **37K** | **33K** | **29K** | **26K** | **23K** | **20K** | **17K** | **15K** | **12K** | **11K** | **8,678** | **7,181** | **5,798** | **4,619** | **3,605** | **2,777** | **2,016** | **1,431** | **993** | **601** | **110** |
| ***N_e_*** | **15** | **18** | **20** | **24** | **27** | **32** | **38** | **45** | **53** | **63** | **76** | **91** | **108** | **135** | **164** | **202** | **254** | **310** | **376** | **465** | **575** | **675** | **743** | **923** | **1,155** |
| **Generation** | **12** | **14** | **16** | **19** | **22** | **26** | **31** | **37** | **44** | **53** | **65** | **79** | **97** | **120** | **149** | **186** | **233** | **292** | **365** | **453** | **551** | **657** | **757** | **844** | **983** |
| **TH** | | | | | | | | | | | | | | | | | | | | | | | | | |
| **Fragment size** | **60K** | **54K** | **49K** | **44K** | **40K** | **36K** | **32K** | **28K** | **25K** | **22K** | **19K** | **16K** | **14K** | **12K** | **9,928** | **8,256** | **6,737** | **5,360** | **4,161** | **3,245** | **2,477** | **1,816** | **1,204** | **793** | **131** |
| ***N_e_*** | **83** | **95** | **112** | **127** | **146** | **172** | **198** | **236** | **269** | **325** | **388** | **447** | **522** | **613** | **695** | **809** | **884** | **1,039** | **1,126** | **1,223** | **1,449** | **1,609** | **1,655** | **1,754** | **2,104** |
| **Generation** | **12** | **14** | **16** | **19** | **22** | **26** | **31** | **37** | **44** | **53** | **65** | **79** | **97** | **120** | **149** | **186** | **233** | **292** | **366** | **453** | **553** | **657** | **758** | **845** | **982** |
| **NEL** | | | | | | | | | | | | | | | | | | | | | | | | | |
| **Fragment size** | **89K** | **81K** | **74K** | **67K** | **60K** | **54K** | **48K** | **42K** | **37K** | **33K** | **28K** | **24K** | **21K** | **18K** | **15K** | **12K** | **10K** | **7,983** | **6,155** | **4,799** | **3,549** | **2,618** | **1,735** | **1,151** | **65** |
| ***N_e_*** | **66** | **76** | **85** | **97** | **111** | **128** | **148** | **170** | **199** | **231** | **274** | **329** | **391** | **458** | **537** | **640** | **756** | **884** | **994** | **1,101** | **1,330** | **1,500** | **1,744** | **1,894** | **2,987** |
| **Generation** | **12** | **14** | **16** | **19** | **22** | **26** | **31** | **37** | **44** | **53** | **65** | **79** | **97** | **120** | **149** | **186** | **233** | **292** | **365** | **453** | **552** | **658** | **757** | **845** | **994** |
| **LM** | | | | | | | | | | | | | | | | | | | | | | | | | |
| **Fragment size** | **240K** | **219K** | **198K** | **179K** | **160K** | **144K** | **127K** | **113K** | **99K** | **86K** | **75K** | **64K** | **55K** | **46K** | **38K** | **32K** | **26K** | **21K** | **16K** | **12K** | **9,279** | **6,628** | **4,479** | **2,986** | **167** |
| ***N_e_*** | **93** | **106** | **121** | **139** | **160** | **184** | **213** | **243** | **287** | **338** | **393** | **460** | **543** | **658** | **770** | **874** | **1,010** | **1,173** | **1,282** | **1,468** | **1,623** | **1,761** | **1,831** | **1,990** | **1,885** |
| **Generation** | **12** | **14** | **16** | **19** | **22** | **26** | **31** | **37** | **44** | **53** | **65** | **79** | **97** | **120** | **149** | **186** | **233** | **293** | **365** | **453** | **552** | **657** | **758** | **845** | **994** |
| **SIM** | | | | | | | | | | | | | | | | | | | | | | | | | |
| **Fragment size** | **230K** | **210K** | **190K** | **171K** | **154K** | **138K** | **122K** | **108K** | **95K** | **83K** | **72K** | **61K** | **52K** | **44K** | **30K** | **25K** | **20K** | **15K** | **12K** | **8,765** | **6,415** | **4,292** | **2,853** | **1,745** | **143** |
| ***N_e_*** | **85** | **96** | **109** | **124** | **141** | **163** | **186** | **215** | **246** | **287** | **337** | **399** | **465** | **554** | **642** | **751** | **893** | **1,023** | **1,170** | **1,303** | **1,496** | **1,632** | **1,676** | **1,970** | **1,542** |
| **Generation** | **12** | **14** | **16** | **19** | **22** | **26** | **31** | **37** | **44** | **53** | **65** | **79** | **97** | **120** | **149** | **186** | **233** | **293** | **366** | **453** | **552** | **658** | **758** | **845** | **994** |
